# Cyclo­hexa­naminium trichloro­acetate

**DOI:** 10.1107/S1600536809017504

**Published:** 2009-05-20

**Authors:** Durre Shahwar, M. Nawaz Tahir, Naeem Ahmad, Muhammad Akmal Khan, Afifa Saeed

**Affiliations:** a Department of Chemistry, Government College University, Lahore, Pakistan; b Department of Physics, University of Sargodha, Sargodha, Pakistan

## Abstract

In the crystal of the title compound, C_6_H_14_N^+^·C_2_Cl_3_O_2_^−^, centrosymmetric assemblies of two cyclo­hexa­naminium cations and two trichloro­acetate ions are linked by N—H⋯O hydrogen bonds, thereby forming *R*_4_^4^(12) ring motifs. Further N—H⋯O inter­actions link the tetra­mers into chains propagating along the *a* axis.

## Related literature

For related structures, see: Shahwar *et al.* (2009 ▶); Wang *et al.* (2005 ▶); Jones & Ahrens (1998 ▶). For reference structural data, see: Allen *et al.* (1987 ▶). For graph-set notation, see: Bernstein *et al.* (1995 ▶).
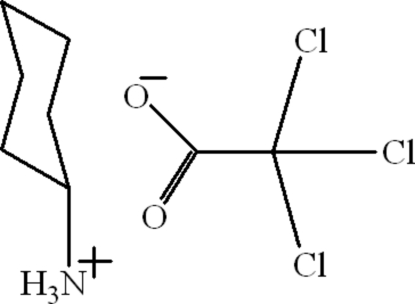


## Experimental

### 

#### Crystal data

C_6_H_14_N^+^·C_2_Cl_3_O_2_^−^
*M* *_r_* = 262.55
Monoclinic, 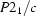

*a* = 6.7217 (4) Å
*b* = 21.2482 (15) Å
*c* = 10.6908 (6) Å
β = 126.590 (3)°
*V* = 1225.98 (14) Å^3^
*Z* = 4
Mo *K*α radiation
μ = 0.72 mm^−1^
*T* = 296 K
0.25 × 0.18 × 0.12 mm

#### Data collection

Bruker Kappa APEXII CCD diffractometer
Absorption correction: multi-scan (*SADABS*; Bruker, 2005 ▶) *T*_min_ = 0.853, *T*_max_ = 0.919
13379 measured reflections
2910 independent reflections
1710 reflections with *I* > 2σ(*I*)
*R*_int_ = 0.038

#### Refinement

*R*[*F* ^2^ > 2σ(*F* ^2^)] = 0.066
*wR*(*F* ^2^) = 0.215
*S* = 1.05
2910 reflections
139 parameters
H atoms treated by a mixture of independent and constrained refinement
Δρ_max_ = 0.73 e Å^−3^
Δρ_min_ = −0.37 e Å^−3^

### 

Data collection: *APEX2* (Bruker, 2007 ▶); cell refinement: *SAINT* (Bruker, 2007 ▶); data reduction: *SAINT*; program(s) used to solve structure: *SHELXS97* (Sheldrick, 2008 ▶); program(s) used to refine structure: *SHELXL97* (Sheldrick, 2008 ▶); molecular graphics: *ORTEP-3 for Windows* (Farrugia, 1997 ▶) and *PLATON* (Spek, 2009 ▶); software used to prepare material for publication: *WinGX* (Farrugia, 1999 ▶) and *PLATON*.

## Supplementary Material

Crystal structure: contains datablocks global, I. DOI: 10.1107/S1600536809017504/hb2970sup1.cif

Structure factors: contains datablocks I. DOI: 10.1107/S1600536809017504/hb2970Isup2.hkl

Additional supplementary materials:  crystallographic information; 3D view; checkCIF report

## Figures and Tables

**Table 1:** Hydrogen-bond geometry (Å, °)

*D*—H⋯*A*	*D*—H	H⋯*A*	*D*⋯*A*	*D*—H⋯*A*
N1—H1*A*⋯O1^i^	0.85 (6)	1.96 (6)	2.788 (6)	167 (4)
N1—H1*B*⋯O2^ii^	0.82 (5)	1.96 (5)	2.770 (5)	168 (4)
N1—H1*C*⋯O1^iii^	1.02 (4)	1.83 (4)	2.837 (4)	169 (4)

Symmetry codes: (i) 
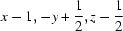
; (ii) 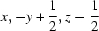; (iii) 
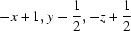
.

**Fig. 1.**
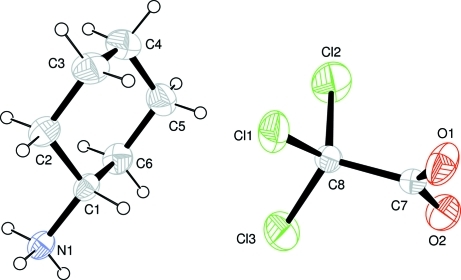
View of (I) with displacement ellipsoids drawn at the 30% probability level. H-atoms are shown by small spheres of arbitrary radius.

**Fig. 2.**
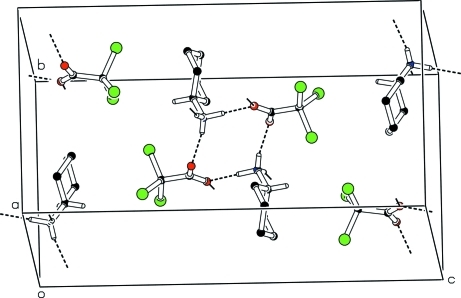
The partial packing in (I) showing intermolecular H-bonding between NH3 and trichloroacetate ions and the resulting ring motif.

## supplementary crystallographic information

### Comment

In continuation of synthesizing various organic ammonium salts (Shahwar *et al.*, 2009), the title compound (I), (Fig. 1) is being reported. The crystal structures of (II) Cyclohexylammonium dichloroacetate (Wang *et al.*, 2005) and (III) Cyclohexylamine cyclohexylammonium chloride (Jones & Ahrens, 1998) have been reported.

In (I), the bond distance and bond angles are within normal ranges (Allen *et al.*, 1987). In the title compound, two cyclohexanaminium ions and two trichloroacetate ions are interlinked through intermolecular H-bonding of N—H···O type (Table 1) forming ring motifs *R*_4_^4^(12) (Bernstein *et al.*, 1995) (Fig. 2). The ring motifs are further connected through the same along the *a* axis resulting in one-dimensional polymeric chains. The cyclohexanaminium ions are in chair confirmations with N-atoms at a distance of 0.628 (9)Å from the central plane.

### Experimental

A solution of trichloroacetic acid (1.635 g, 0.01 mol) in 20 ml of dichloromethane was prepared. To this solution cyclohexyl amine (1.14 ml, 0.01 mol) was added dropwise and stirred for 30 min. The precipitate were filtered out and recrystallized in hot chloroform to yield colourless rods of (I).

### Refinement

The coordinates of H-atoms attached to  N1 and C1 were refined. The other H atoms were positioned geometrically (C—H = 0.97 Å) and refined as riding.  The constraint U_iso_(H) = 1.2U_eq_(carrier) was applied for all H atoms.

### Crystal data

**Table:**

C_6_H_14_N^+^·C_2_Cl_3_O_2_^−^	*F*(000) = 544
*M**_r_* = 262.55	*D*_x_ = 1.422 Mg m^−^^3^
Monoclinic, *P*2_1_/*c*	Mo *K*α radiation, λ = 0.71073 Å
Hall symbol: -P 2ybc	Cell parameters from 2910 reflections
*a* = 6.7217 (4) Å	θ = 2.6–27.9°
*b* = 21.2482 (15) Å	µ = 0.72 mm^−^^1^
*c* = 10.6908 (6) Å	*T* = 296 K
β = 126.590 (3)°	Rod, colorless
*V* = 1225.98 (14) Å^3^	0.25 × 0.18 × 0.12 mm
*Z* = 4	

### Data collection

**Table:**

Bruker Kappa APEXII CCD diffractometer	2910 independent reflections
Radiation source: fine-focus sealed tube	1710 reflections with *I* > 2σ(*I*)
graphite	*R*_int_ = 0.038
Detector resolution: 7.50 pixels mm^-1^	θ_max_ = 27.9°, θ_min_ = 2.6°
ω scans	*h* = −8→8
Absorption correction: multi-scan (*SADABS*; Bruker, 2005)	*k* = −27→26
*T*_min_ = 0.853, *T*_max_ = 0.919	*l* = −12→14
13379 measured reflections	

### Refinement

**Table:**

Refinement on *F*^2^	Primary atom site location: structure-invariant direct methods
Least-squares matrix: full	Secondary atom site location: difference Fourier map
*R*[*F*^2^ > 2σ(*F*^2^)] = 0.066	Hydrogen site location: inferred from neighbouring sites
*wR*(*F*^2^) = 0.215	H atoms treated by a mixture of independent and constrained refinement
*S* = 1.05	*w* = 1/[σ^2^(*F*_o_^2^) + (0.1089*P*)^2^ + 0.5776*P*] where *P* = (*F*_o_^2^ + 2*F*_c_^2^)/3
2910 reflections	(Δ/σ)_max_ < 0.001
139 parameters	Δρ_max_ = 0.73 e Å^−^^3^
0 restraints	Δρ_min_ = −0.37 e Å^−^^3^

### Special details

**Table:**

Geometry. Bond distances, angles *etc*. have been calculated using the rounded fractional coordinates. All su's are estimated from the variances of the (full) variance-covariance matrix. The cell e.s.d.'s are taken into account in the estimation of distances, angles and torsion angles
Refinement. Refinement of *F*^2^ against ALL reflections. The weighted *R*-factor *wR* and goodness of fit *S* are based on *F*^2^, conventional *R*-factors *R* are based on *F*, with *F* set to zero for negative *F*^2^. The threshold expression of *F*^2^ > σ(*F*^2^) is used only for calculating *R*-factors(gt) *etc*. and is not relevant to the choice of reflections for refinement. *R*-factors based on *F*^2^ are statistically about twice as large as those based on *F*, and *R*- factors based on ALL data will be even larger.

### Fractional atomic coordinates and isotropic or equivalent isotropic displacement parameters (Å^2^)

**Table:**

	*x*	*y*	*z*	*U*_iso_*/*U*_eq_	
N1	0.2904 (5)	0.05714 (15)	0.0547 (4)	0.0480 (10)	
C1	0.4574 (6)	0.08854 (16)	0.2093 (4)	0.0463 (10)	
C2	0.7215 (6)	0.06696 (19)	0.2878 (5)	0.0568 (11)	
C3	0.8922 (7)	0.0967 (2)	0.4465 (5)	0.0731 (15)	
C4	0.8100 (8)	0.0827 (3)	0.5472 (6)	0.0816 (18)	
C5	0.5457 (8)	0.1032 (2)	0.4704 (5)	0.0764 (17)	
C6	0.3726 (7)	0.0733 (2)	0.3100 (5)	0.0626 (15)	
Cl1	0.9294 (2)	0.30601 (6)	0.38465 (15)	0.0791 (4)	
Cl2	0.7598 (3)	0.28803 (6)	0.57059 (16)	0.1029 (6)	
Cl3	0.4167 (2)	0.28226 (6)	0.23280 (17)	0.0950 (5)	
O1	0.7893 (5)	0.42783 (12)	0.4227 (4)	0.0711 (9)	
O2	0.4297 (5)	0.40429 (14)	0.3696 (3)	0.0680 (10)	
C7	0.6272 (5)	0.39152 (15)	0.3948 (3)	0.0406 (9)	
C8	0.6778 (7)	0.32022 (15)	0.3937 (4)	0.0488 (10)	
H1	0.432 (7)	0.1331 (19)	0.186 (4)	0.0553*	
H1A	0.141 (8)	0.0678 (19)	0.011 (5)	0.0575*	
H1B	0.333 (7)	0.0632 (19)	−0.002 (5)	0.0575*	
H1C	0.285 (7)	0.010 (2)	0.071 (4)	0.0575*	
H2A	0.72989	0.02151	0.29833	0.0680*	
H2B	0.77440	0.07841	0.22401	0.0680*	
H3A	1.05899	0.08085	0.49682	0.0877*	
H3B	0.89524	0.14188	0.43499	0.0877*	
H4A	0.91813	0.10433	0.64607	0.0973*	
H4B	0.82413	0.03787	0.56795	0.0973*	
H5A	0.53487	0.14865	0.46040	0.0919*	
H5B	0.49516	0.09095	0.53505	0.0919*	
H6A	0.20568	0.08903	0.25991	0.0753*	
H6B	0.37023	0.02808	0.32080	0.0753*	

### Atomic displacement parameters (Å^2^)

**Table:**

	*U*^11^	*U*^22^	*U*^33^	*U*^12^	*U*^13^	*U*^23^
N1	0.0401 (14)	0.0439 (17)	0.0601 (19)	0.0011 (12)	0.0300 (14)	0.0040 (13)
C1	0.0436 (16)	0.0341 (17)	0.057 (2)	0.0007 (13)	0.0278 (16)	0.0035 (14)
C2	0.0426 (17)	0.063 (2)	0.066 (2)	0.0015 (16)	0.0331 (17)	0.0043 (18)
C3	0.0463 (19)	0.084 (3)	0.072 (3)	−0.0074 (19)	0.026 (2)	−0.002 (2)
C4	0.066 (2)	0.093 (4)	0.064 (3)	−0.002 (2)	0.027 (2)	−0.007 (2)
C5	0.079 (3)	0.088 (3)	0.068 (3)	0.003 (2)	0.047 (2)	−0.010 (2)
C6	0.0508 (19)	0.072 (3)	0.074 (3)	−0.0019 (18)	0.042 (2)	−0.001 (2)
Cl1	0.0779 (7)	0.0700 (7)	0.0894 (8)	0.0142 (5)	0.0499 (7)	−0.0143 (6)
Cl2	0.1635 (14)	0.0733 (8)	0.0817 (9)	0.0174 (8)	0.0784 (10)	0.0311 (6)
Cl3	0.0748 (7)	0.0673 (8)	0.0937 (9)	−0.0196 (5)	0.0236 (7)	−0.0294 (6)
O1	0.0498 (14)	0.0401 (14)	0.111 (2)	−0.0005 (11)	0.0413 (15)	0.0006 (14)
O2	0.0564 (15)	0.0762 (19)	0.0787 (19)	0.0110 (13)	0.0442 (15)	0.0000 (14)
C7	0.0409 (16)	0.0400 (17)	0.0342 (15)	0.0016 (13)	0.0187 (13)	0.0004 (12)
C8	0.0579 (19)	0.0354 (17)	0.0418 (17)	−0.0009 (14)	0.0236 (16)	0.0005 (13)

### Geometric parameters (Å, °)

**Table:**

Cl1—C8	1.776 (6)	C5—C6	1.523 (6)
Cl2—C8	1.762 (4)	C1—H1	0.97 (4)
Cl3—C8	1.758 (4)	C2—H2B	0.9700
O1—C7	1.218 (5)	C2—H2A	0.9700
O2—C7	1.217 (5)	C3—H3A	0.9700
N1—C1	1.492 (5)	C3—H3B	0.9700
N1—H1A	0.85 (6)	C4—H4A	0.9700
N1—H1C	1.02 (4)	C4—H4B	0.9700
N1—H1B	0.82 (5)	C5—H5B	0.9700
C1—C6	1.523 (7)	C5—H5A	0.9700
C1—C2	1.513 (7)	C6—H6B	0.9700
C2—C3	1.508 (6)	C6—H6A	0.9700
C3—C4	1.504 (8)	C7—C8	1.554 (5)
C4—C5	1.510 (9)		
			
C1—N1—H1C	109 (2)	H3A—C3—H3B	108.00
C1—N1—H1A	111 (3)	C3—C4—H4A	109.00
C1—N1—H1B	113 (3)	C3—C4—H4B	109.00
H1B—N1—H1C	110 (4)	C5—C4—H4A	109.00
H1A—N1—H1B	112 (5)	C5—C4—H4B	109.00
H1A—N1—H1C	102 (4)	H4A—C4—H4B	108.00
N1—C1—C2	109.8 (3)	C4—C5—H5A	110.00
N1—C1—C6	109.7 (4)	C4—C5—H5B	109.00
C2—C1—C6	110.7 (3)	C6—C5—H5A	110.00
C1—C2—C3	110.6 (4)	C6—C5—H5B	109.00
C2—C3—C4	111.4 (4)	H5A—C5—H5B	108.00
C3—C4—C5	111.6 (4)	C1—C6—H6A	109.00
C4—C5—C6	110.7 (4)	C1—C6—H6B	110.00
C1—C6—C5	110.5 (4)	C5—C6—H6A	109.00
N1—C1—H1	105 (2)	C5—C6—H6B	110.00
C2—C1—H1	114 (3)	H6A—C6—H6B	108.00
C6—C1—H1	108 (3)	O1—C7—O2	127.7 (3)
C1—C2—H2A	110.00	O1—C7—C8	116.8 (4)
C1—C2—H2B	110.00	O2—C7—C8	115.5 (3)
C3—C2—H2A	110.00	Cl1—C8—Cl2	107.1 (2)
C3—C2—H2B	110.00	Cl1—C8—Cl3	107.2 (2)
H2A—C2—H2B	108.00	Cl1—C8—C7	112.7 (3)
C2—C3—H3A	109.00	Cl2—C8—Cl3	111.3 (2)
C2—C3—H3B	109.00	Cl2—C8—C7	107.5 (2)
C4—C3—H3A	109.00	Cl3—C8—C7	111.1 (3)
C4—C3—H3B	109.00		
			
N1—C1—C2—C3	−178.3 (3)	C4—C5—C6—C1	−55.8 (5)
C6—C1—C2—C3	−57.1 (4)	O1—C7—C8—Cl1	−15.1 (4)
N1—C1—C6—C5	178.2 (3)	O1—C7—C8—Cl2	102.6 (4)
C2—C1—C6—C5	56.9 (4)	O1—C7—C8—Cl3	−135.5 (3)
C1—C2—C3—C4	56.5 (5)	O2—C7—C8—Cl1	165.9 (2)
C2—C3—C4—C5	−55.9 (6)	O2—C7—C8—Cl2	−76.4 (3)
C3—C4—C5—C6	55.4 (6)	O2—C7—C8—Cl3	45.6 (4)

### Hydrogen-bond geometry (Å, °)

**Table:**

*D*—H···*A*	*D*—H	H···*A*	*D*···*A*	*D*—H···*A*
N1—H1A···O1^i^	0.85 (6)	1.96 (6)	2.788 (6)	167 (4)
N1—H1B···O2^ii^	0.82 (5)	1.96 (5)	2.770 (5)	168 (4)
N1—H1C···O1^iii^	1.02 (4)	1.83 (4)	2.837 (4)	169 (4)

Symmetry codes: (i) *x*−1, −*y*+1/2, *z*−1/2; (ii) *x*, −*y*+1/2, *z*−1/2; (iii) −*x*+1, *y*−1/2, −*z*+1/2.
